# Shadow AI and the Limits of Institutional Agility

**DOI:** 10.2196/112827

**Published:** 2026-10-14

**Authors:** Benjamin Rader, John S Brownstein

**Affiliations:** 1 Innovation and Digital Health Accelerator, Boston Children's Hospital, 300 Longwood Ave, Boston, MA, 02115, United States, 1 6173551389; 2 Harvard Medical School, Boston, MA, United States

**Keywords:** generative artificial intelligence, large language models, clinical decision support, clinician workarounds, health system governance, medical device regulation, regulatory sandbox

## Abstract

Physicians are adopting generative AI tools faster than health systems can evaluate them. This commentary on the empirical report of “Shadow AI” drivers by Petersson and colleagues argues that governing Shadow AI requires health systems to pair institutional speed with transparent, stakeholder-engaged governance.

Clinical AI models are rapidly improving and, as Petersson and colleagues describe [1], clinicians’ interest in the use of these tools has outpaced hospitals’ ability to govern them. Large language models optimized for diagnostic reasoning have already exceeded clinicians on evaluations of complex, real-world case reports [2]. Clinicians and health systems professionals are moving quickly to harness these capabilities. However, implementation often proceeds faster than institutions or regulators can catch up, leaving early adopters to test and validate these tools in a vacuum [3]. Petersson and colleagues discuss this uptake of Shadow AI and provide empirical evidence explaining why the use of AI tools that lack institutional sanction and regulatory authorization has emerged [1].

Their qualitative analysis of 357 physicians in Sweden identified four primary purposes for Shadow AI use: (1) clinical decision support, (2) administrative tasks, (3) research, and (4) technological exploration. Many physicians access these capabilities through personal devices and accounts via tools like ChatGPT. As Petersson and colleagues find, these tools compensate for gaps in institutional systems, reduce cognitive workload, and provide access to knowledge that formal channels make difficult to reach. Shadow AI appears to have taken off despite the fact that many of these tools are not authorized as medical devices under the European Union (EU) Medical Device Regulation.

The adoption of Shadow AI follows an established pattern in health care. It has long been clinical practice to prescribe off-label treatments when the perceived evidence outpaces the label or when looking for an edge over what is currently available, a convention that is frequently applied to new technologies [4]. In the past, clinicians have used personal smartphones to photograph wounds and share them over messaging apps before secure image-sharing infrastructure existed [4]. Documentation support technologies such as mobile rounding and handoff applications were also adopted by clinicians long before hospitals officially integrated them into the electronic health record [4].

Petersson and colleagues summarize the purposes of Shadow AI as largely individual, including the pursuit of “rapid productivity gains” and the chance to “experiment with cutting-edge technology” [1]. While potentially effective at meeting these objectives, individual needs can often contrast with the broader goals of health systems. A clinician is focused on treating the patient in front of them, whereas health systems must serve entire populations over decades, which depends on sustained community trust. Rapid deployment of any technology without transparency can be harmful. A single highly publicized failure or adverse event can make a treatment or technology unpalatable for years. Additionally, the utilization of Shadow AI introduces legal risk to the health system around both the privacy of protected health information and the use of unapproved technology that may not have demonstrated clinical validity [3].

Public sentiment regarding generative AI in health care is also becoming increasingly polarized [5], and past technological innovations show that even an effective technology will have low community buy-in if introduced without due consideration of all stakeholders. Consider the United Kingdom’s Federated Data Platform, for which NHS England awarded Palantir Technologies a multiyear contract [6]. NHS England attributes substantial operational gains to the platform, including approximately 110,000 additional patients treated in hospital theaters, a 15% reduction in discharge delays overall, and a 36% reduction in average delay days for long-stay patients [7]. Public acceptance has not followed suit. Over roughly 4 months in mid-2026, roughly 60,000 patients withdrew their records through the national data opt-out, a surge the Minister for Health Innovation linked to press coverage of Palantir’s role in the program [6]. In July 2026, a House of Commons committee recommended that the government prepare to drop the platform when the contract’s break clause opens in February 2027 [8]. The platform’s purported operational benefits have not produced the public trust its long-term deployment requires.

In the past, we have argued that innovation pathways should follow clinicians’ lead to bring Shadow AI into a safe and secure environment [3]. While this remains true, institutional agility is not a universal remedy and even well-resourced hospitals struggle to wrangle Shadow AI at the rate new tools appear. Within the EU, software intended for diagnosis or treatment requires conformity assessment under the Medical Device Regulation. In the United States, the Food and Drug Administration offers a premarket pathway for software as a medical device. AI tools intended for diagnosis or treatment generally require 510(k) clearance, De Novo authorization, or premarket approval before they can be marketed for clinical use [9]. However, these requirements are shifting. The mandate for EU member states to operate national AI regulatory sandboxes by August 2027 provides a way to validate high-risk systems under official supervision [10]. In the United States, no federal mandate yet parallels the EU’s requirement and comparable sandbox programs are only just emerging at the state level.

Governance must move beyond prohibition to account for the tension between AI’s risks and benefits, a tension that clinicians are already weighing for themselves [1]. Institutional speed and regulatory pathways address only one side of this tension, and trust is the other. Shadow AI currently sits in a space of low institutional speed and low trust (Table 1), and increasing speed alone will not close this gap. Agility must be combined with transparency, regulatory legitimacy, and broad stakeholder engagement to bring Shadow AI into a space of long-term sustainability.

**Table 1.** A framework for governing Shadow AI adoption by institutional speed and trust investment.

	Low trust investment	High trust investment
Slow institutional response	Status quo. Shadow AI persists. Physicians quietly route around governance to unauthorized tools. Unmet institutional need and unmonitored risk accumulate together.	Stalled adoption. Governance is transparent and well-intentioned but too slow to meet clinical need. Informal workarounds continue despite good-faith efforts.
Fast institutional response	Backlash risk. Tools reach practice quickly without regulatory legitimacy or clear communication. Operational benefit does not guarantee acceptance (eg, the NHS Federated Data Platform).	Target. Trusted, sanctioned adoption. Institutional speed is paired with regulatory sandbox validation, transparent governance, clinician and patient engagement. The pull toward Shadow AI diminishes as sanctioned tools meet clinical needs.
